# Intertrochanteric Nailing Without a Traction Table: A Practical Positioning Technique on a Standard Operating Table

**DOI:** 10.7759/cureus.98970

**Published:** 2025-12-11

**Authors:** Kamil Balaban

**Affiliations:** 1 Orthopedics and Traumatology, Ministry of Health Finike State Hospital, Antalya, TUR

**Keywords:** intertrochanteric fracture, intertrochanteric hip fracture, intramedullary nailing, operating rooms, operating theatre, patient positioning

## Abstract

Intertrochanteric femur fractures often require cephalomedullary nailing, yet many centers, particularly those in resource-limited settings, lack traction tables. This technical note introduces a novel positioning method that enables safe and reproducible intramedullary fixation on a standard radiolucent operating table with a central post, a configuration for which practical guidance is limited in the literature. The technique uses caudal table translation, 15-20° ipsilateral sacral elevation, and 40-50° contralateral limb lowering to achieve adequate anteroposterior and lateral fluoroscopic visualization without specialized equipment. This approach is intended for orthopedic surgeons working in hospitals with restricted infrastructure, offering improved ergonomics, reliable imaging, and efficient workflow. Potential challenges, including limited leg support, fluoroscopic overlap, and contamination risk during under-table imaging, can be minimized with appropriate preparation. The method provides a practical alternative for centers without traction tables and expands operative feasibility in equipment-constrained environments.

## Introduction

Geriatric hip fractures, particularly unstable intertrochanteric femur fractures, constitute an increasing workload for orthopedic surgeons as life expectancy rises. Intramedullary nailing has become the preferred treatment option owing to its biomechanical advantages, relatively minimally invasive nature, and favorable functional outcomes [1]. Successful intertrochanteric fracture fixation, however, depends not only on implant choice and fracture reduction but also on appropriate patient positioning [2].

Optimal positioning improves surgical exposure, facilitates anteroposterior (AP) and lateral fluoroscopic imaging, and supports accurate implant placement while minimizing operative time and complication risk [3]. Dedicated radiolucent traction tables, which allow independent manipulation of both lower extremities and provide sustained traction, are therefore widely recommended for intertrochanteric nailing. At the same time, these tables are associated with prolonged setup, traction-related nerve injury, technical difficulties in obese patients, and substantial acquisition and maintenance costs [4,5]. Access to traction tables remains limited in many low- and middle-income countries and in smaller hospitals [6,7].

Alternative positioning techniques, such as supine without a traction table, supine semilithotomy, and lateral decubitus positions, have been described for the surgical treatment of intertrochanteric fractures [2,3,8,9]. The alternative patient positions were documented with acceptable reductions and alignment, emphasizing that the choice should primarily depend on the surgeon's familiarity. In most reports, radiolucent tables without a central post were used, or details of the table configuration were not clearly specified. In daily practice, however, one of the most commonly available operating tables is the standard modular radiolucent table with a centrally positioned post. This table is routinely used for hip and knee arthroplasty as well as distal extremity fracture surgery; however, it is generally not preferred for the fixation of hip or pelvic fractures because the central post obstructs proximal access for the C-arm and prevents rotation beneath the table, thereby limiting the ability to obtain adequate AP and lateral fluoroscopic views [10].

There is limited guidance on how to adapt intertrochanteric nailing to this standard table configuration while preserving intraoperative imaging quality and surgeon ergonomics. Such guidance may be particularly valuable in hospitals that lack traction tables or radiolucent tables without a central post but still encounter a high volume of hip fractures.

This technical note describes a practical positioning technique that enables cephalomedullary nailing of unstable intertrochanteric femur fractures on a standard radiolucent operating table with a central post. The technique is simple, reproducible, and designed to be applicable in resource-limited operating rooms. Its advantages, limitations, and potential pitfalls are discussed.

## Technical report

An 87-year-old female patient presented with an unstable right intertrochanteric femur fracture classified as AO Foundation/Orthopaedic Trauma Association (AO/OTA) 31-A3.3 following a low-energy fall. Cephalomedullary nailing was planned. After routine preoperative evaluation, spinal anesthesia was administered, and 1 g of intravenous cefazolin was given prior to incision.

Patient positioning and table configuration

The procedure was performed on a standard radiolucent operating table with a central post. To facilitate C-arm access to the proximal femur, the table was slid to its most caudal position until the central post lay just proximal to the patient’s ipsilateral iliac crest. This maneuver positioned the pelvis slightly distal to the post and created additional space around the hip region.

A folded sheet or gel pad was placed under the ipsilateral sacrum, elevating it by approximately 15-20°. This elevation opened the space between the pelvis and the contralateral hip and helped reduce overlap of bony structures during fluoroscopy. Care was taken to ensure that the patient remained stable on the table despite the sacral elevation.

The contralateral leg was positioned in the leg holder and lowered approximately 40-50° relative to the plane of the table. This configuration prevented the contralateral thigh from obscuring the operative field or interfering with the C-arm and aiming device. The operative limb was maintained close to neutral rotation and alignment to facilitate subsequent reduction maneuvers.

The ipsilateral arm was gently flexed at the elbow, placed across the chest, and secured with arm holders or soft straps to avoid interference with the insertion point and instrumentation. The contralateral arm was kept off the table on a separate arm board as per the anesthesiologist’s preference. Standard safety checks, including assessment of pressure points and neurovascular status, were performed.

C-arm positioning and imaging

**Figure 1:**
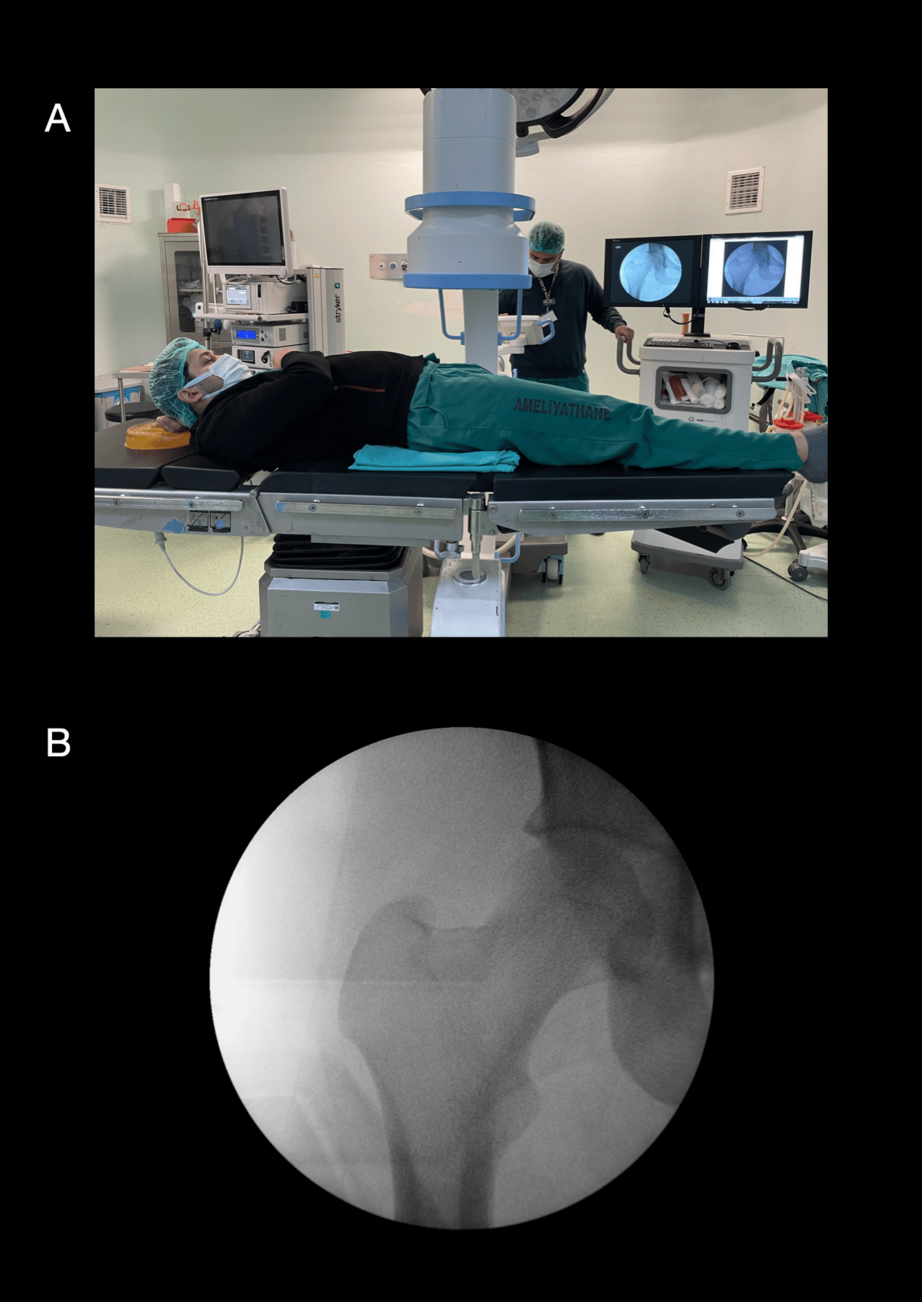
Patient positioning and anteroposterior fluoroscopic imaging (A) The patient is positioned supine on a standard radiolucent operating table that has been moved caudally, allowing the pelvis to rest distal to the central post. A folded sheet elevates the ipsilateral sacrum approximately 15–20°. (B) Anteroposterior fluoroscopic image showing unobstructed visualization of the femoral head, neck, and trochanteric region.

The C-arm was introduced from the contralateral side of the table to obtain anteroposterior (AP) views of the proximal femur. The image intensifier was positioned to provide an accurate AP projection centered on the femoral head and neck (Figure 1).

**Figure 2:**
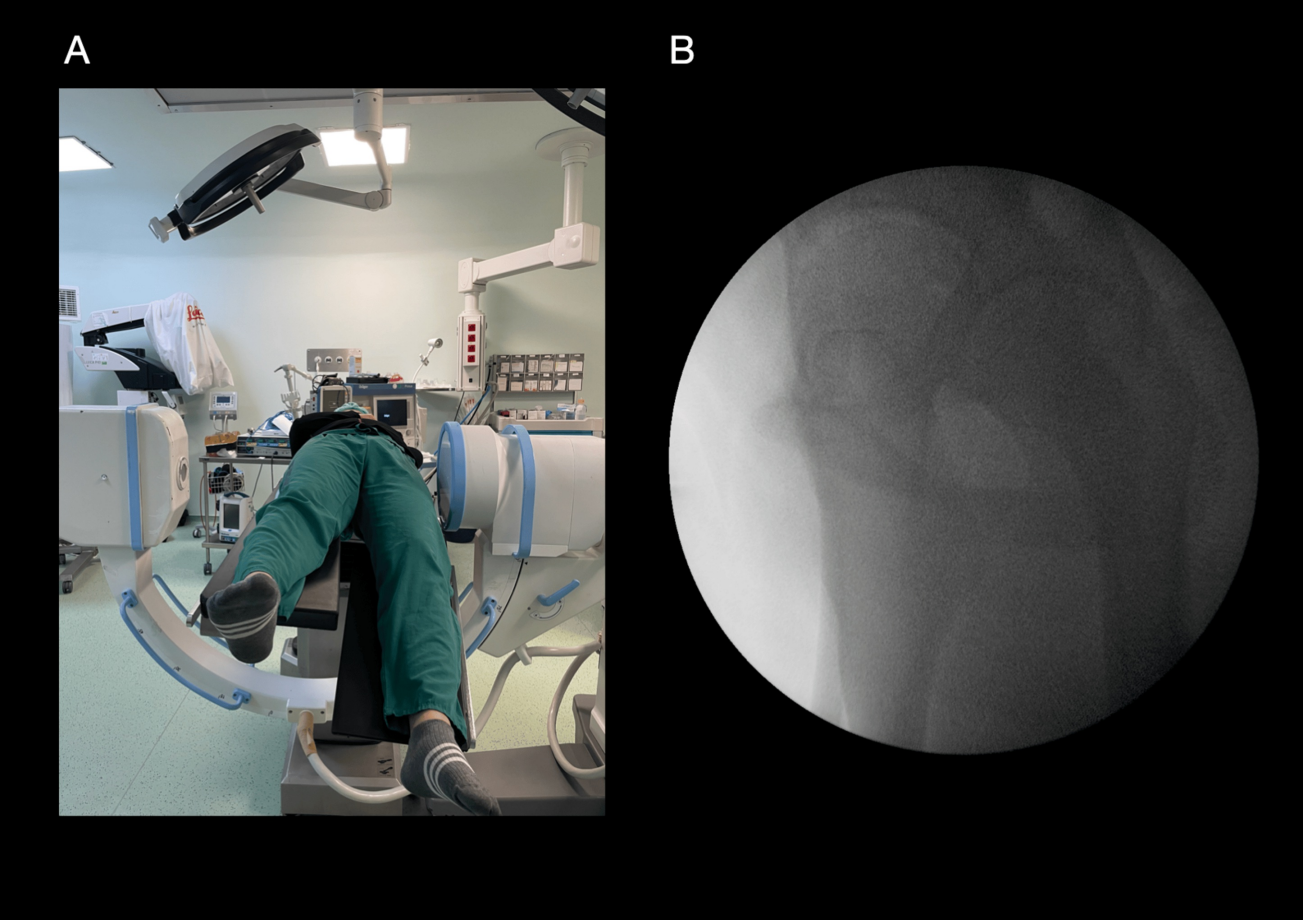
Patient positioning and lateral fluoroscopic imaging (A) The contralateral lower extremity was lowered approximately 40–50° using the leg positioner to prevent thigh overlap. (B) True lateral fluoroscopic view obtained by advancing the C-arm beneath the operating table.

For lateral imaging, the C-arm was passed carefully under the table and rotated approximately 90° around the hip center until an accurate lateral view of the proximal femur was obtained (Figure 2).

A barrier of sterile drapes and towels was placed along the path of the C-arm to minimize the risk of contamination when the device was advanced under the drapes. If the trochanteric region or implant instruments appeared partially superimposed by the contralateral hip or by the table rails during lateral imaging, a modest table tilt of approximately 5-10° toward the contralateral side was applied. Fluoroscopy was repeated after each adjustment to confirm improved visualization.

Fracture reduction and intramedullary nailing

Closed reduction was achieved by longitudinal manual traction and manipulation of the operative limb, assisted by an assistant standing at the foot of the table. Traction was applied intermittently rather than continuously, and fracture alignment was monitored fluoroscopically. When necessary, a percutaneous clamp was introduced through a small anterior incision to improve reduction and maintain alignment.

Once an acceptable reduction had been obtained, the entry point was identified at the tip of the greater trochanter under fluoroscopy. A guidewire was inserted, followed by the opening of the trochanteric entry site using the manufacturer’s reamer. A cephalomedullary nail system (Proximal Femoral Nail, ASES Medikal, Gaziantep, Turkey) was inserted without routine diaphyseal reaming in accordance with the implant protocol.

**Figure 3:**
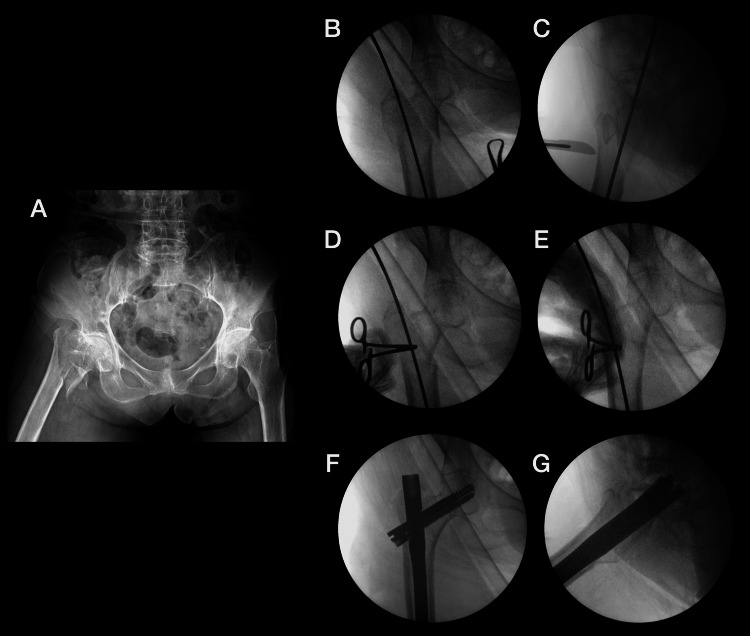
Sequential fluoroscopic assessment of intertrochanteric fracture fixation on a standard table (A) Preoperative anteroposterior radiograph of an 87-year-old female patient showing a right-sided unstable intertrochanteric femur fracture (AO/OTA 31-A3.3). (B, C) Intraoperative anteroposterior and lateral images confirm the identification of the entry point and preliminary alignment. (D, E) Closed reduction was performed using longitudinal traction and anterior percutaneous clamping, confirmed by fluoroscopy. (F, G) Final anteroposterior and lateral fluoroscopic images showing satisfactory reduction, restoration of a neck–shaft angle near 130°, and an acceptable tip–apex distance (15–25 mm) following cephalomedullary nail insertion. AO/OTA: AO Foundation/Orthopaedic Trauma Association

Proximal lag and compression screws were then placed in the femoral neck and head under fluoroscopic guidance. The aim was to maintain a neck-shaft angle of approximately 130° and to achieve a tip-apex distance (TAD) between 15 and 25 mm in both AP and lateral views. Distal locking was performed through the jig according to the standard technique. Final AP and lateral fluoroscopic images confirmed satisfactory fracture reduction, appropriate implant position, and acceptable TAD (Figure 3).

At the end of the procedure, the surgical field was irrigated, the incisions were closed in layers, and a sterile dressing was applied. The patient was transferred to the recovery unit. Postoperative rehabilitation and weight-bearing protocols were tailored based on the patient’s overall condition.

## Discussion

This technical note describes a modified supine positioning technique that allows cephalomedullary nailing of unstable intertrochanteric femur fractures on a standard radiolucent operating table with a central post. The technique adapts commonly available operating room equipment to the requirements of hip fracture fixation without the need for a dedicated traction table.

The most commonly preferred approach for intramedullary nailing in unstable intertrochanteric fractures is the supine position on a traction table; this configuration offers advantages because it allows for controlled long-axis traction, a relatively standardized fluoroscopy setup, and safe airway management for the anesthesia team [11,12]. However, the supine-traction table combination has been questioned due to equipment requirements, lengthy setup time, risk of traction-related nerve injury, and difficulty positioning in obese patients, leading to the emergence of alternative positions such as lateral decubitus, semilithotomy, and supine without traction [13]. Studies and meta-analyses examining the use of cephalomedullary nails in the lateral decubitus position report that this approach can be performed with similar or shorter operative times, less blood loss, and similar complication rates compared to the supine-traction table [14,15]. Consequently, it can provide significant technical advantages in terms of alignment of the entry point and reamer-nail trajectory, especially in obese or polytrauma patients.

Recent comparative studies assessing the supine position with a traction table, the supine position without traction, and the lateral decubitus position have reported heterogeneous findings, and some have demonstrated a relative advantage of traction table use in terms of reduction quality, lag-screw positioning accuracy, tip-apex distance, and collodiaphyseal alignment [16,17]. Accordingly, the available evidence does not support a clear equivalence among all positioning methods. Rather, each technique presents a distinct profile of strengths and limitations. In settings where a traction table is unavailable, the supine position without traction may still yield acceptable reduction and complication rates when meticulous fluoroscopic control and adjunctive maneuvers are employed; however, certain parameters, particularly collodiaphyseal angle control, may be inferior to traction-table-assisted reductions, as reported in prior series [12,18]. Although the supine semilithotomy position has been proposed to enhance lateral visualization, this approach carries drawbacks, including groin pressure, technical difficulty in obtaining and maintaining the position, and increased personnel requirements [19]. Accordingly, positioning choice should be individualized based on institutional resources, surgeon experience, and patient-specific considerations rather than an assumption of equivalent radiological outcomes across techniques.

Alternative techniques using supine, semilithotomy, or lateral decubitus positions have been reported as feasible options for intertrochanteric nailing without the use of a traction table [3,13,20]. However, most of these techniques assume the use of a radiolucent table without a central post or do not describe the table configuration in enough detail. The presence of a central post can hinder proximal access for the C-arm, making it difficult to obtain an adequate fluoroscopic image. It may discourage surgeons in resource-limited hospitals, where the only available option is a standard modular radiolucent table with a central post, from attempting hip fracture fixation on such tables.

The present technique addresses this gap by combining three simple maneuvers--caudal translation of the table, elevation of the ipsilateral sacrum, and controlled lowering of the contralateral leg--to create a working corridor around the proximal femur. This configuration reduces overlap between the contralateral limb, the central post, and the aiming device, thereby improving both AP and lateral fluoroscopic views. The method can be implemented quickly with minimal additional equipment and may reduce preparation time compared to setting up the traction table. In addition, it preserves the familiar anatomical orientation of the supine position and allows the surgical team to perform manual traction and reduction maneuvers as needed.

Limitations and pitfalls

The technique has several limitations and potential pitfalls that are mainly related to patient habitus and imaging constraints. In tall patients, sliding the table caudally may result in both lower extremities extending beyond the distal end of the table, leading to insufficient leg support and difficulty maintaining traction. Inadequate support can cause inadvertent limb movement, compromise fracture reduction, or prolong operative time. To mitigate this risk, additional leg supports or padded stools should be prepared in advance in coordination with the anesthesia team.

Despite ipsilateral sacral elevation and contralateral leg lowering, partial superimposition of the fracture site by the contralateral hip or by the metal side rails of the table may persist, particularly during lateral imaging. Poor visualization may hinder accurate assessment of fracture reduction, neck-shaft angle, and TAD, increasing the risk of malpositioned implants and subsequent mechanical failure. Routine pre-draping fluoroscopic checks are therefore recommended to confirm that both AP and lateral views are satisfactory before finalizing the draping and positioning. When necessary, a modest table tilt of 5-10° toward the contralateral side can improve visualization, but excessive tilt should be avoided because it may alter fracture alignment or inadvertently change femoral rotation. Intraoperative confirmation of rotational alignment using the lesser trochanter profile and comparison with the contralateral side is essential.

Another potential pitfall is inadequate stabilization of the ipsilateral arm or sacral support. If the arm is not securely positioned across the chest, it may interfere with nail insertion or risk iatrogenic injury during manipulation. Similarly, a poorly positioned or unstable sacral support can shift during the operation, altering pelvic position and compromising reduction. Proper fixation of the arm and secure placement of the folded sheet or gel pad are, therefore, critical for maintaining a stable setup.

Passing the C-arm under the table to obtain lateral views introduces a risk of contamination of the sterile field. Without preventive measures, contact between the C-arm and the drapes may increase the likelihood of surgical-site infection. To address this, sterile drapes and towel barriers should be placed along the expected path of the C-arm before it arrives. The surgical team should be vigilant for any breaks in sterility and ready to adjust the drapes if necessary.

Key technical pearls and common pitfalls associated with this approach are summarized in Table 1.

**Table 1:** Pearls and pitfalls of the positioning strategy for proximal femoral nailing on standard radiolucent operating tables

Pearls	Pitfalls
Iliac crest positioned distal to the central post	Protruding lower extremity from the table in tall patients
Folded sheet support under the ipsilateral sacrum	Too much lateral positioning of the hip overlapping with the lateral table rails
Ipsilateral arm secured on the chest	Consider increasing the trunk tilt
Lowering the contralateral leg	Inspection of hip and pelvic rotation
Performing manual traction and reduction	Additional sterile towels for lateral views

Clinical implications and future perspectives

Despite these limitations, the described technique offers a practical solution for performing intertrochanteric nailing in operating rooms where traction tables are unavailable. Relying on a standard table with a central post--equipment that is already present in many institutions--may lower the threshold for providing operative treatment to frail, elderly patients with unstable fractures. Furthermore, the technique preserves the advantageous biomechanics of cephalomedullary nailing while avoiding the costs and logistic challenges associated with traction tables.

Future work could include larger case series evaluating operative time, fluoroscopy duration, reduction quality, and complication rates associated with this positioning method compared with traditional traction table techniques. Such data would help clarify whether the described approach can achieve equivalent or superior outcomes while remaining accessible to resource-limited centers.

## Conclusions

The described modified supine positioning technique enables cephalomedullary nailing of unstable intertrochanteric femur fractures on a standard radiolucent operating table with a central post, without the need for a dedicated traction table. By combining caudal translation of the table, modest elevation of the ipsilateral sacrum, and controlled lowering of the contralateral leg, the method provides satisfactory AP and lateral fluoroscopic views and preserves surgeon ergonomics. It is simple, reproducible, and widely applicable, particularly in resource-limited operating rooms where traction tables or radiolucent tables without a central post are not available. Careful attention to leg support, imaging quality, and maintenance of sterility is essential to minimize pitfalls and optimize surgical outcomes.
